# Prospects for detecting signs of life on exoplanets in the JWST era

**DOI:** 10.1073/pnas.2416188122

**Published:** 2025-09-22

**Authors:** Sara Seager, Luis Welbanks, Lucas Ellerbroek, William Bains, Janusz J. Petkowski

**Affiliations:** ^a^Department of Earth, Atmospheric and Planetary Sciences, Massachusetts Institute of Technology, Cambridge, MA 02139; ^b^Department of Physics, Massachusetts Institute of Technology, Cambridge, MA 02139; ^c^Department of Aeronautical and Astronautical Engineering, Massachusetts Institute of Technology, Cambridge, MA 02139; ^d^School of Earth and Space Exploration, Arizona State University, Tempe, AZ 85287; ^e^Department of Astrophysics/Institute for Mathematics, Astrophysics and Particle Physics, Radboud University, Nijmegen 6500 GL, The Netherlands; ^f^School of Physics and Astronomy, Cardiff University, Cardiff CF24 3AA, United Kingdom; ^g^JJ Scientific, Warsaw, Mazowieckie 02-792, Poland; ^h^Faculty of Environmental Engineering, Wroclaw University of Science and Technology, Wroclaw 50-370, Poland

**Keywords:** exoplanets, astrobiology, biosignatures, planetary atmospheres

## Abstract

The search for signs of life in the Universe has entered a new phase with the advent of the James Webb Space Telescope (JWST). Detecting biosignature gases via exoplanet atmosphere transmission spectroscopy is in principle within JWST’s reach. We reflect on JWST’s early results in the context of the potential search for biological activity on exoplanets. The results confront us with a complex reality. Established inverse methods to interpret observed spectra—already known to be highly averaged representations of intricate three dimensional (3D) atmospheric processes—can lead to disparate interpretations even with JWST’s quality of data. Characterizing rocky or sub-Neptune-size exoplanets with JWST is an intricate task, and moves us away from the notion of finding a definitive “silver bullet” biosignature gas. Indeed, JWST results necessitate us to allow “parallel interpretations” that will perhaps not be resolved until the next generation of observatories. Nonetheless, with a handful of habitable-zone planet atmospheres accessible given the anticipated noise floor, JWST may continue to contribute to this journey by designating a planet as biosignature gas candidate. To do this we will need to sufficiently refine our inverse methods and physical models for confidently quantifying specific gas abundances and constraining the atmosphere context. Looking ahead, future telescopes and innovative observational strategies will be essential for the reliable detection of biosignature gases.

The quest to understand life beyond Earth is one as old as humanity itself. Since the earliest days of modern astronomy, the presence of oxygen (O_2_) in Earth’s atmosphere was recognized as due to, and hence to be a signature of life (1). Oxygen appeared in Earth’s atmosphere at least 2.7 billion years ago (2) when primitive cyanobacteria evolved to perform photosynthesis, converting carbon dioxide (CO_2_) and water (H_2_O) into carbohydrates and oxygen, using sunlight. This process changed the chemical composition of Earth’s atmosphere by introducing O_2_.

From an exoplanetary perspective, Earth’s 20% atmospheric oxygen is anomalous due to oxygen’s high reactivity, sustained by oxygenic photosynthesis. An extraterrestrial civilization with advanced telescopic technologies observing Earth, could interpret O_2_’s high concentration as a strong indicator of life.

Oxygen is the paradigmatic example of a “biosignature gas,” a gas that is produced by life and accumulates to high enough atmospheric abundances to be detected with remote telescopes. Yet despite evidence pointing to the very early origin of oxygenic photosynthesis 3.5 Gya (3), O_2_ took billions of years to accumulate to its present-day values of 20% even while O_2_-producing life was present (4). Therefore, during much of the time when we are confident that Earth was inhabited, O_2_ was at most a trace component of the atmosphere.

Molecules other than O_2_ should therefore be considered as potential biosignature gases. Earth’s biosphere generates thousands of different volatile molecules for various reasons—as waste products from exploiting chemical potential energy gradients, for signaling or defense against predators, to name a few—which could be mirrored or replaced by different compounds on other worlds. Indeed, aside from the noble gases, every gas in Earth’s atmosphere to part-per-trillion levels is produced by biological activity (5), although most gases have a primary source other than life. Extensive exploration of different gases, not just those produced in significant quantities by life on Earth, is needed.

We now have our first real opportunity to search for exoplanet atmosphere biosignature gases with the recently operational James Webb Space Telescope [JWST; (6)]―a search that is becoming an expanding area of research within astrobiology. We aim to assess the present opportunities and challenges to using the JWST to search for exoplanet atmosphere signs of life. We begin with a review of JWST’s enabling photometric precision as it relates to target planet numbers (Section 1) and continue with prospects and pitfalls via current exoplanet atmosphere interpretation methods (Section 2). We directly discuss the prospects for JWST to detect biosignature gases (Section 3), and conclude with future needs including innovative methods and new telescopes (Section 4).

## 1. JWST Transmission Spectroscopy Precision and Habitable-Zone Targets

**Fig. 1.**
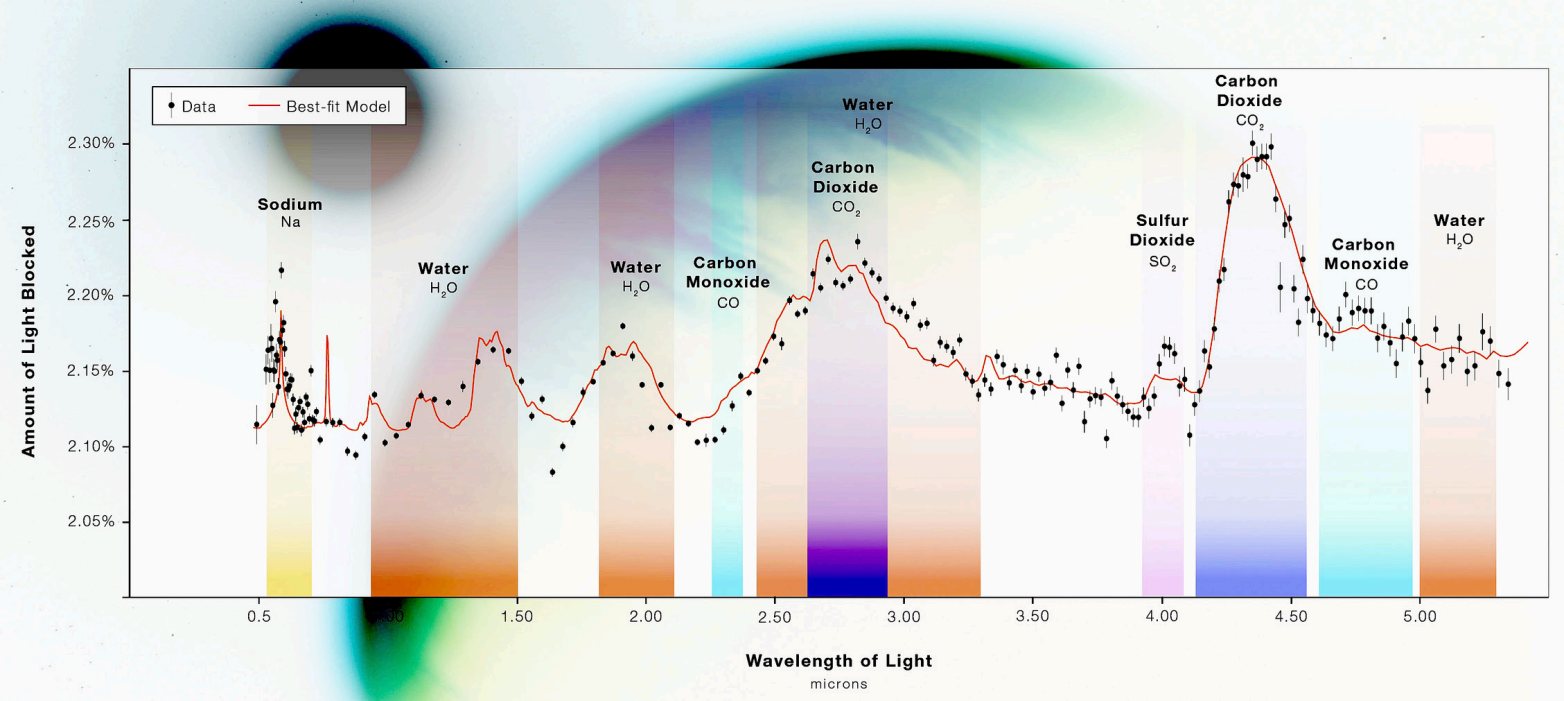
Wasp 39b transmission spectrum. The planet size (shown as amount of light blocked) changes with wavelengths, increasing where individual gases are strongly absorbing. Image credit: NASA, ESA, CSA, STScI.

To understand the prospect of detecting biosignature gases with JWST we summarize a defining JWST observation related to its data precision and noise floor, and estimate what this noise floor translates into for numbers of targets suitable for biosignature gas searches. JWST uses transmission spectroscopy (7, 8), the dimming of a star as a planet transits across its face, allowing us to analyze the starlight filtered through the planet’s atmosphere (Fig. 1). This method reveals variations in atmospheric composition across different wavelengths due to selective absorption by atmospheric gases. Other JWST atmosphere observation methods do not reach habitable-zone temperate exoplanets.^*^ Such a planet’s low infrared emission will be overshadowed by radiation from their host stars, making them unsuitable for secondary eclipse spectroscopy, and additionally their small planet-star separations make them unsuitable for direct imaging.

Among JWST’s many notable observations, we highlight an early, striking demonstration of JWST’s unprecedented precision, leading to the unequivocal, robust detection of carbon dioxide (CO_2_) in the atmosphere of the “hot Saturn” exoplanet WASP-39b [M_p_ = 0.28 M_J_, R_p_ = 1.27 R_J_, P = 4.1 d, T_eq_ ~ 1,100 K; Fig. 1; (9)]. The detection of CO_2_ is striking because CO_2_’s spectral band is clearly visible “by eye” in the data. Despite not being the dominant carbon-bearing species in WASP-39b’s hydrogen-dominated atmosphere, CO_2_, strongly absorbs infrared light, creating a distinct spectral feature. Sulfur dioxide (SO_2_), also detected in WASP-39b’s atmosphere, arises from photochemical reactions rather than predicted chemical equilibrium (10). This highlights the complexities of exoplanet atmospheres, and marks a shift toward a new subfield of astrochemistry. We emphasize that WASP-39b is a giant exoplanet too hot for life beneath its massive hydrogen-helium envelope.

**Fig. 2.**
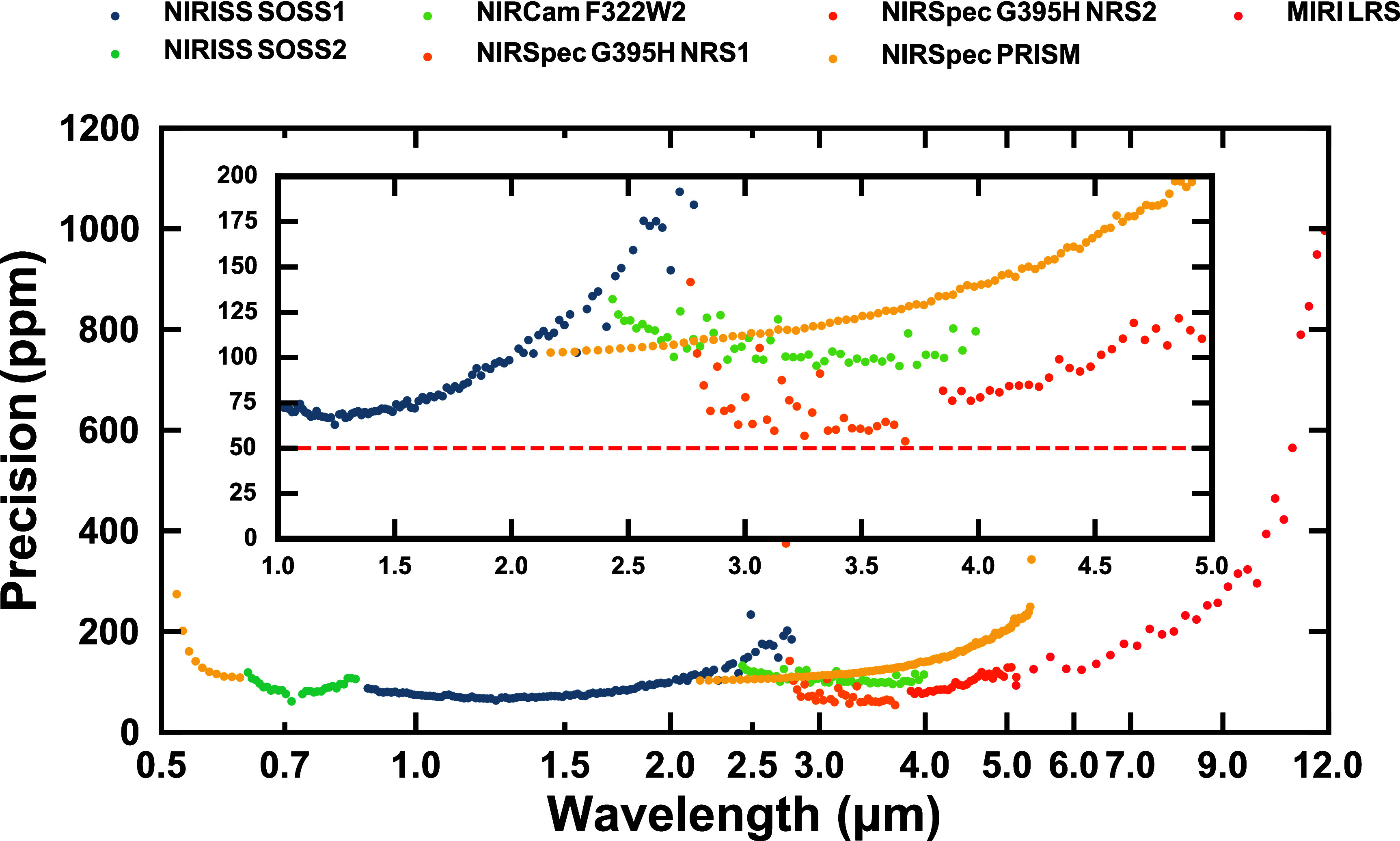
Spectroscopic photometric precision of WASP-39b. The precision (*y*-axis) as a function of wavelength (*x*-axis) for different JWST instruments (colors, as noted in the legend). As an Early Release Science Program target, WASP-39b was observed with each JWST instrument, NIRISS SOSS (0.6 to 2.8 μm), NIRSpec G395H (2.7 to 5.2 μm), NIRSpec PRISM (0.5 to 5.5 μm), NIRCam F322W2 (2.0 to 4.0 μm), and MIRI-LRS (5.0 to 12.0 μm). While the precision in the measured transit depth is chromatic and dependent on the stellar spectrum, the spectrum of WASP-39b shows precisions below 200 ppm between 1.0 and 5.0 μm. Not shown is that the spectroscopic light curve data precision is close to the photon-limited noise (9). Data from ref. 14 accounting for precision inflation.

With one transit for the relatively bright star WASP-39, JWST has achieved 50 ppm precision (Fig. 2), demonstrating performance of the telescope at the spectroscopic light curve close to the photon-limited noise (9). The 50 ppm value is similar to that achieved by other observational programs for sub-Neptunes and super-Earths (11–13). JWST’s true noise floor can only be assessed with several combined transits, to disentangle photon noise from actual instrumental limitations. Although this assessment is ongoing, we adopt a 30 ppm noise floor value for the purposes of this review.

By noise floor, we mean the minimum level of unwanted signal arising from a combination of factors related to the instrument and observational environment (e.g., thermal fluctuations, mechanical vibrations, characteristics of the infrared detectors, and unknown sources) (15, 16). This variety of contributing factors makes the noise floor best determined empirically.

We can qualitatively investigate what kind, and how many, exoplanets in their host star’s habitable zone are accessible under the 30 ppm noise floor. We estimate the transmission spectroscopy signal (*TS*), by taking the area of a three scale height (*H*) tall atmosphere annulus compared to the area of a homogeneous background star.

[1] $$TS\approx \frac{6HR_{p}}{R_{*}^{2}}.$$

Here *R_p_* and R_*_ are the planet and star radius respectively. *H* is defined as H=kT/mg, where *k* is Boltzmann’s constant, *T* is temperature, *m* is mean molecular mass, and *g* is surface gravity.

We can immediately rule out the JWST transmission spectroscopy study of the atmosphere of an Earth-like planet in an Earth-like orbit around a Sun-like star from Eq. **1**. Namely, Earth-like planets’ small sizes (*R_p_* ~ 6,400 km) and thin atmospheres (*H* = 8 km) are impractical for transmission spectroscopy against the backdrop of a Sun-sized star (R_*_ ~ 700,000 km). Here *TS* ~ 1 ppm—far lower than the adopted 30 ppm noise floor.

The observational favorability of M dwarf stars can also be seen from Eq. **1**. Since M dwarf stars are half to one tenth of the size of our Sun, the *TS* will have signals 4 to 100 times larger than Sun-sized star hosts.

We can further use the approximate *TS* feasibility criteria to estimate the number of habitable-zone exoplanets with accessible atmospheres. Considering the list of currently observed or planned exoplanet targets from the JWST Cycles 1 through 3 from the Transiting Exoplanets List of Space Telescope Spectroscopy Catalog (TrExoLiSTS) (15) and estimating the surface temperature by the equilibrium temperature (*T*_eq_) with a cutoff of 300 K for habitable-zone planets, we find only a few Earth-size planets (*R_p_* ≤ 1.5 *R_Earth_*), namely some of the Trappist-1 exoplanets and LP 791-18d. (Here *T*_eq_ is the effective temperature attained by an isothermal planet atmosphere after it has reached complete equilibrium with the radiation from its parent star.

Sub-Neptunes are of interest in the search for life not necessarily because they are expected to be habitable, but because their likely H_2_-dominated atmospheres with scale heights 14 times larger than N_2_-dominated atmospheres make them more favorable for atmospheric observations. Some sub-Neptunes are hypothesized to have liquid water under the right conditions (16, 17), needed for life as we know it. Regardless of the presence of water oceans, some sub-Neptunes may have liquid water clouds, offering the potential for an aerial biosphere (18).

As many as half a dozen sub-Neptune-sized exoplanets (1.5 *R_Earth_* ≤ *R_p_* ≤ 3 *R_Earth_*) meet our *TS* feasibility criteria, even with a conservative *T_eq_* cutoff of 373 K (100 °C). However, these planets are also likely too hot for life due to greenhouse effects from H_2_, collision-induced absorption (19). Observational biases from K2’s and TESS’s viewing segments have favored shorter orbital periods, but ongoing TESS observations should uncover sub-Neptunes with longer periods and therefore cooler temperatures.

**Fig. 3.**
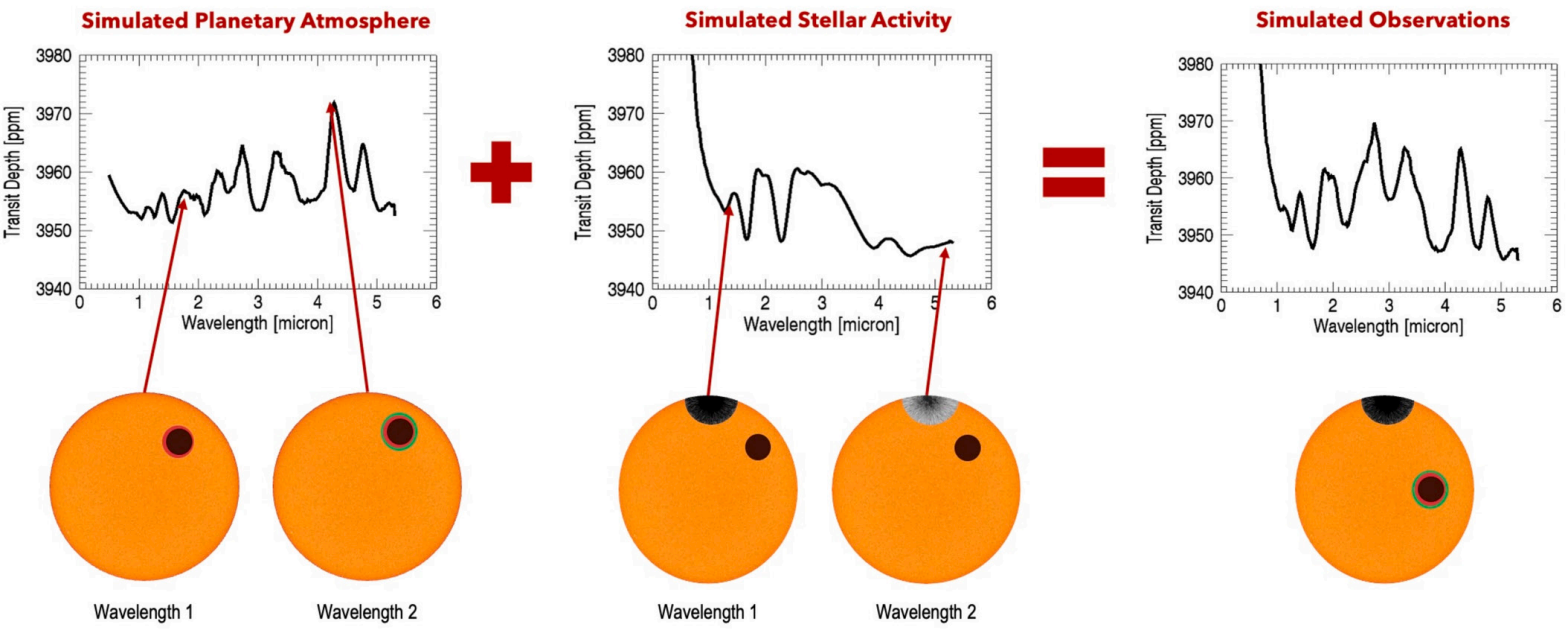
Star spot contamination in transit transmission spectra. *Left*: Transit depth varies with wavelength as a planet (black circle) transits a star (orange disk) with an atmosphere (colored annuli), due to wavelength-dependent atmospheric absorption. *Middle*: A star spot (black/gray semicircle) alters the star’s apparent size with wavelength, affecting the transit depth even for a planet without an atmosphere. *Right*: The combined spectrum shows contributions from both the planet’s atmosphere and the star spot, complicating interpretation. Image credit: Seager and Shapiro.

Although M dwarf stars are the only accessible temperate planet-hosting targets for JWST, M dwarf stars present a challenge. Their stellar magnetic activity, higher than for Solar-type stars, manifests as star spots, faculae, and flares that contaminate the spectra (20). Star spots can mimic transit transmission spectra by the star’s inhomogeneity (Fig. 3). In the case of Trappist-1, the measured star contamination dominates the signal (12). The community is intensively pursuing many ideas to mitigate or remove contamination signals (20). Some stochastic variability might never be adequately modeled and may just have to be accepted as part of the noise floor.

## 2. From Spectroscopy Measurements to Planetary Characterization

To understand the JWST’s prospects for identifying biosignature gases, we begin by examining ongoing planet characterization. We unpack the process of translating observations into estimates of exoplanet properties, beginning with a general description, then proceeding to a case study on sub-Neptunes, and concluding with the relevance for biosignature gases.

It may seem a stretch to use spectra to ascertain planetary properties (atmosphere abundances, surface and interior bulk composition, habitability and presence of life, and more). After all, observed exoplanet spectra represent a highly averaged signal of complex 3D physical and chemical atmospheric processes, reduced to relative changes in the observed wavelength dependence of the combined star and planet light as a point source. We live with a family of potential solutions for planet properties that fit the current imperfect and incomplete data. To quantify the range of planet properties, the community resorts to atmosphere retrieval (21), an umbrella term for a subset of inverse methods. This process determines the underlying atmospheric parameters that best match the observations for a given model (see e.g., the review by ref. 22). The outcome is not only an assessment of how well a specific model fits, but also a statistical measure of the likely values for the model’s parameters, such as probable abundances of atmospheric gases.

**Fig. 4.**
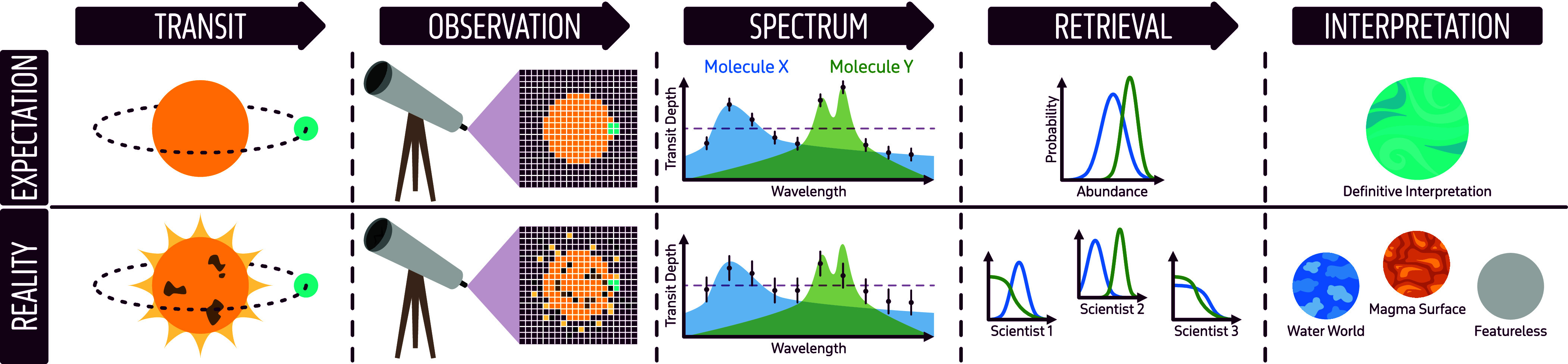
Illustrative schematic of the atmospheric data analysis and interpretation process, from observations through interpretation of an exoplanet’s nature. The pathway involves four main steps: 1] taking observations; 2] processing the data from pixels to a spectrum; 3] interpreting the resulting spectrum with model fits to infer planetary properties such as gas abundances; and 4] drawing conclusions about the planet properties. The *Top* row is inspired by WASP-107b (24) a relatively straightforward case, and the *Bottom* row is motivated by TOI 270d (25), a disputed planetary nature. All panels are illustrative only. See text for details. Image credit: Seager, Welbanks, Tilke.

Inferring planet properties from spectroscopic measurements is not straightforward as the process involves a number of steps with subjective choices (Fig. 4). Step 1 is the telescope observations (i.e., raw pixel data). Step 2 is converting the observations into a planetary spectrum via a data analysis pipeline, which always involves a number of assumptions. Step 3 is producing the family of models that fit the data—inferring the underlying planet atmosphere properties (i.e., temperature, gas abundances) from the data. Step 3 is challenging due to the large number of free parameters in most models compared to the number of data points, and the ensuing degeneracies between these parameters. These challenges cause atmospheric retrieval to be highly sensitive to the choice of model parameterization and model assumptions (see e.g., ref. 23), and in some cases to small changes in the data and the structure of the data uncertainties. This can result in inferred planet properties that are ultimately incorrect—and rejected by standard frequentist metrics such as chi-squared statistics and *P*-value hypothesis testing. Moreover, most atmospheric retrievals rely on the assumption of Gaussian uncorrelated noise, whereas the data retrieved on have known correlated systematic noise. Step 4—planetary interpretation—is not only the end goal but also the motivation for studying exoplanet atmospheres. Step 4 includes identifying the planet archetype (e.g., “water world” or “lava planet”), habitability, presence of biosignature gases, and more. Unfortunately, Step 4 is the most subjective in the entire process of exoplanetary characterization, and is still evolving. In some cases, the uncertainties arising from Steps 1 to 3 are so significant that they cannot rule out fundamentally different planet archetypes. In other situations, the planet’s atmosphere is simply unrevealing of its nature. Unveiling the “truth” of a planet with incomplete and imperfect data and models is the ultimate test of interpolation and extrapolation, yet a challenge we must embrace to characterize exoplanets.

To illustrate the challenges faced by the atmospheric data analysis and interpretation framework let us turn to examples of sub-Neptunes. Sub-Neptunes defy straightforward classification because their average densities match a variety of bulk interiors. They could be water worlds with 50% or more water by mass with liquid water (or supercritical water) ocean; scaled-down versions of Neptune with a thick H_2_-He envelope overlaying a layer of hot dense water plasma over a rocky core; they could be predominantly composed of silicates, but with a mixed hydrogen envelope possibly overlying a magma ocean (e.g., ref. 26); or even scenarios we have yet to consider. The community has aimed to use atmosphere measurements to sort through the possibilities for sub-Neptune archetypes.

A classic example of the challenges in every step in the data-to-characterization process is the case of K2-18b (M_p_ = 8.6 M_E_, R_p_ = 2.6 R_E_, P = 32.94 d, T_eq_ ~ 260 K) (27, 28). Hubble Space Telescope (HST) observations led to a detection of atmospheric water vapor in the atmosphere of K2-18b (29, 30). Yet, later observations with the JWST found no water vapor, and attributed the same atmospheric spectral feature to CH_4_ (17). Furthermore, the nondetection of NH_3_ in K2-18b’s atmosphere was presented as a case for a Hycean world (a planet with a liquid water ocean covered with a H_2_-dominated atmosphere), since NH_3_ is highly soluble in liquid water thus explaining its absence (17). This conclusion was later disputed, as nitrogen compounds are also highly soluble in magma, and therefore the lack of NH_3_ could signify presence of a magma ocean, not a water ocean (31). Therefore, it is important to recognize that the challenges in Step 2 to 3 have cascading consequences all the way to the fourth and final step—the end goal of planet characterization.

A second example is the sub-Neptune exoplanet TOI-270d (M_p_ = 4.8 M_E_, R_p_ = 2.2 R_E_, P = 11.38 d, T_eq_ ~ 350 K) (32). The same observed pixels from JWST (Step 1) were processed by independent teams (Step 2). Both teams agree that the signal in the data is robust, and that the spectral features are attributed to CH_4_ and H_2_O. The inferred abundances CH_4_ and H_2_O, however, were different between the teams (Step 3). These differences, though small (1 to 2σ) led to inconsistent interpretations of the planetary interior archetype (Step 4). One interpretation is a water-dominated planet with a hot surface water ocean (25), another a massive, mixed atmosphere of H_2_, H_2_O, and CH_4_, overlaying a rocky massive interior (33).

The TOI-270d observations are also a cautionary tale for the JWST future of biosignature gas detection: the identification of both CH_4_ and H_2_O still led to conflicting interpretations of planet archetype. Methane has been championed as a promising biosignature gas for JWST rocky planets—but CH_4_’s biosignature status can only be inferred from the presence of other gases, such as the simultaneous presence of CO_2_ in an atmosphere but little CO (34). Presently, low SNR data yield differences in quantified gas abundances of a factor ten to 100 or higher, so obtaining the necessary abundance constraints, key for establishing the CH_4_ as a biosignature gas, may not be possible. We do, however, echo the argument in favor of CH_4_ as a biosignature gas contender on rocky planets without H_2_-dominated envelopes, due to its ease of detectability at JWST wavelengths (35) and its difficulty being produced in high quantities by known abiotic sources (for an oxidized mantle).

Further relevant to biosignature gases is that biosignature gases almost certainly produce weak signals, as they would originate from thin atmospheres, and likely have low abundances. In cases of low-SNR data with weak or statistically insignificant features, retrieval techniques are often employed to claim detections. A “detection” is sometimes quantified by performing a model comparison between a reference model including the specific parameter being investigated (e.g., a given biosignature gas) and a model excluding the parameter of interest—as a pair of nested models. The model used as reference to claim a detection may be unphysical, may not fit the data appropriately, and may have other parameters with degeneracies relative to the parameter of interest. To recap, the oversensitivity to small changes in the data, the potential trap of comparing two incorrect models, and an opaque definition for the term detection mean that using atmospheric retrieval can cause erroneous reports of detection of biosignature gases (36).

Let us step back and ask whether there are any straightforward cases of observations leading to definitive interpretation. Indeed, the case for the warm Neptune WASP-107b (M_p_ = 0.096 M_J_, Rp = 0.96 R_J_, P = 5.72 d, T_eq_ ~ 750 K) (37) presents such an example. Using our framework in Fig. 4, WASP-107b was observed using JWST’s NIRCam (24) and NIRSpec (38) instruments (Step 1). Data were processed using different independent analysis pipelines (Step 2) and model fits via atmospheric retrieval agreed upon the assessed CH_4_’s abundance. The inferred chemical abundances and temperature structure (Step 3), even with the small differences present in Step 2 independent analysis, indicate that the planet’s atmosphere is in a state of chemical disequilibrium, influenced by photochemistry and, likely, tidal heating (Step 4).

WASP-107b exemplifies a straightforward case because it excels in three key criteria critical to robust planet characterization: detection, attribution, and interpretation (see Section 3 for further details). The planet’s high SNR data ensures a robust detection of spectral features (Key Criterion 1: detection). Multiple spectral features of CH_4_ were confidently attributed to the correct chemical species using different instruments and independent pipelines, which strengthens confidence in the association between the spectral features and the gases responsible for causing them (Key Criterion 2: attribution). Finally, the derived planetary properties—such as chemical abundances, temperature structure, and state of chemical disequilibrium—are consistent across independent analyses, allowing for a confident interpretation of the planet’s atmospheric state (Key Criterion 3: interpretation). These three criteria are essential for any atmospheric investigations, particularly those aimed at identifying biosignature gases, where precision in each of the three areas will be paramount.

Future exoplanet observations may not be as clear-cut as the WASP-107b example presented above. Even in this seemingly straightforward case with strong SNR across broad bands leading to high confidence in the presence of CH_4_, and hence of chemical disequilibrium in the atmosphere, the results do not identify the driver of that disequilibrium. The best path forward is one of cautious optimism, acknowledging the limitations of our methods and data while striving to maximize the insights they offer. Regarding model interpretation, the complexity of the problem often exceeds the fidelity of even JWST data. Prioritizing high SNR features before delving into parameter inference will be essential for reliable interpretations. Simultaneously, advancing models and establishing physical “guardrails” are crucial steps to mitigate the subjective nature of the interpretation exercise. Perhaps for any “biosignature gas candidates,” we may hope for a situation similar to WASP-107b: an atmosphere highly out of chemical equilibrium (not due to UV-driven photochemistry), recognizable as such even under varying model assumptions.

## 3. Biosignature Gas Prospects with JWST

There are three Key Criteria for definitive exoplanet findings, that are also relevant for biosignature gases.

1. Detection: Is the signal robust?
  • For biosignature gases, the robustness of an unambiguous signal detection is highly relevant as any candidate signals are almost certainly going to be weak due to tiny atmospheres on rocky exoplanets and low anticipated atmospheric abundances.
2. Attribution: Are the spectral features correctly attributed to the appropriate gas(es)?
  • For biosignature gases, this involves identifying a distinctive spectral signature that stands out from dominant background atmospheric gases and that is unequivocally attributable to the candidate gas.
3. Interpretation: How reliable are the derived planetary properties?
  • For biosignature gases, this includes ensuring that the detected signal is not confounded by false positives (*SI Appendix*, Table S2) and corresponds to plausible production rates.

In this section, we review current thinking on false positives and biosignature gas production rates (Section 3.1). We next present the growing list of biosignature gases, considering false positives and production rates (Section 3.2). We conclude with a review of the tentative claim of a JWST biosignature gas detection, as confronted against the above three Key Criteria (Section 3.3).

### 3.1. A Framework for Evaluating Potential of Biosignature Gases Detection.

**Fig. 5.**
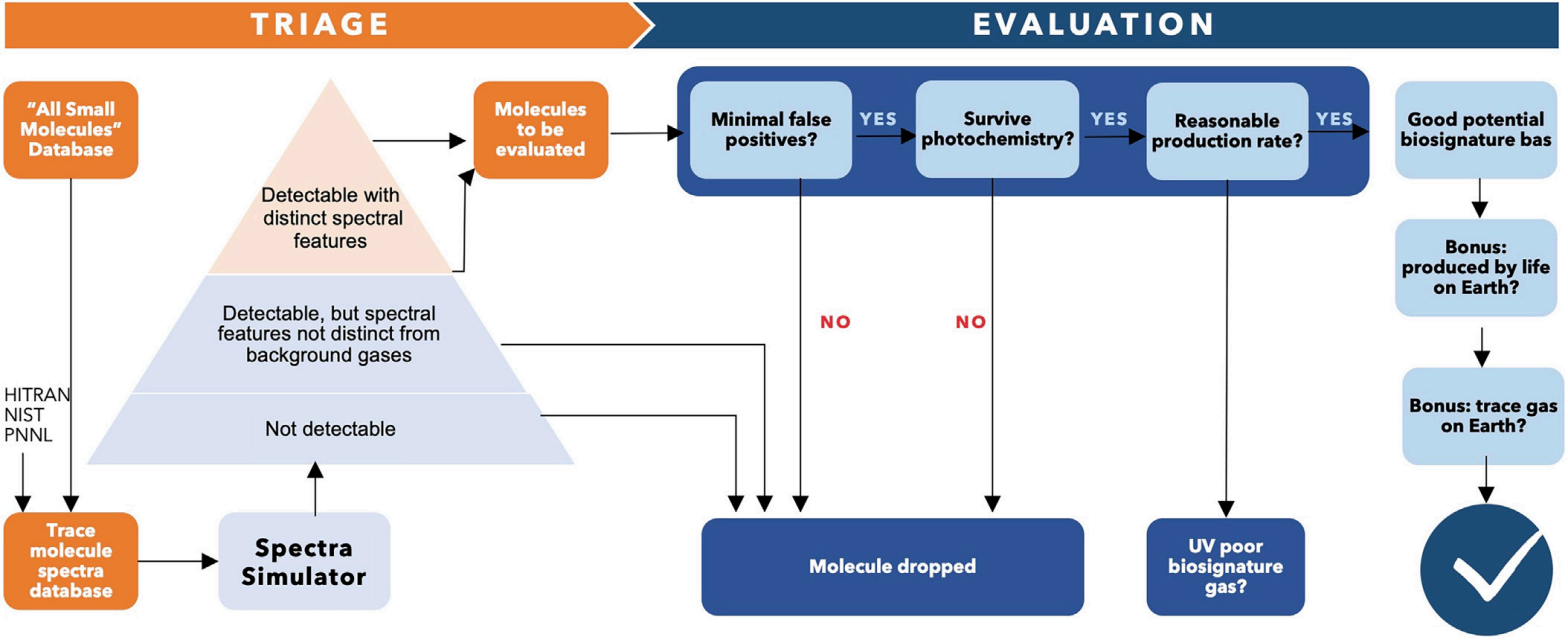
Schematic of a biosignature gas evaluation framework for thousands of gases. The first “triage” step identifies gases with prominent, distinctive spectral features. The second “evaluation” step focuses on photochemistry, assessing a molecule’s atmospheric survival and whether biological production rates, compared to Earth’s biofluxes, are plausible. Image credit: Seager and Zhan.

Ahead of observations, the community can make a list of promising biosignature gases by systematically assessing the potential of thousands of gases known to exist [(5); Fig. 5]. The process begins by determining whether a gas of interest has prominent and distinctive spectral features that stand out from anticipated background atmospheric gases (related to ease of detection and attribution). This is followed by considering potential false positives and, primarily, photochemical stability (both related to interpretation).

#### 3.1.1. Biosignature gas false positives.

Fundamental to the interpretation of biosignature gases is the exclusion of false positives, that is gases that can be produced by abiotic processes as well as by life. Carbon dioxide (CO_2_) is an obvious example, because although ubiquitously produced by life on Earth, CO_2_ is also a significant background atmosphere gas, produced by volcanic processes and atmospheric photochemistry, and is therefore a dominant carbon species in planetary atmospheres. Molecular oxygen (O_2_), despite being a favored biosignature gas, may have an abiotic source from evaporating oceans, originating from a runaway greenhouse effect (39), especially for a planet with close-to-host-star orbit or low mantle FeO and H_2_O inventories (40). For a review of O_2_ and its false positives, see ref. 41. One may estimate a potential biosignature gas’s false positive propensity by a thermodynamic assessment of one or a pair of gases due to volcanic activity. (See the CH_4_ vs. CO_2_ vs. CO example in Section 2).

One concept of robust identification against false positives is that of chemical disequilibrium—the simultaneous presence of reduced and oxidized species in the planet’s atmosphere. The disequilibrium of Earth’s O_2_ and CH_4_ has been considered for decades as a template for life’s expression (42). Methane is speculated to have been present in abundances high enough to warm Earth against the faint young Sun (43). However, the O_2_ and CH_4_ pair is unlikely to be detectable by JWST due to challenges of detecting O_2_ (spectra feature at too short of a wavelength) and O_3_, which is a proxy for O_2_ (low instrument sensitivity at longer wavelengths). Further opposition to atmospheric chemical disequilibrium as a sign of life is the realization that such disequilibrium is a feature of most planets though the degree of chemical disequilibrium is variable. Last, in some cases, chemical disequilibrium may indicate the absence of life, as chemotrophic life forms utilize and diminish thermodynamic disequilibria in their environment, suggesting that disequilibrium could imply no life is present to exploit it. For example, methanotrophic bacteria take up CH_4_ and O_2_ and release CO_2_, thus reducing the CH_4_ (or O_2_) levels in their environment to negligible levels.

Ideally, the community would develop highly sophisticated computer models to assess and even rule out false positives, by tracing a wide variety of gases through extensive planet evolution and ongoing interior and atmosphere processes. The presence of trace gases depends on specific planetary conditions that involve a layer of profound complexity and significant uncertainty (e.g., ref. 44). A large number of unknown parameters accompany the vast array of relevant geophysical processes, including: volcanic outgassing from—and ingassing to—planetary mantles; mantle evolution; mantle convection; magmatic outgassing; atmospheric escape; crustal oxidation; continental and sea floor weathering; deep volatile cycling; ongoing chemical segregation between a planetary core, mantle, and atmosphere, as well as surface, atmospheric, and cloud nonequilibrium photochemical processes (e.g., ref. 26). One effort uses a Monte Carlo approach to sample a wide range of unknown parameters and initial conditions to connect magma ocean crystallization to temperate geochemical cycling (45). The overall complexity is problematic and adds heavily to the model inference problem by increasing the number of free parameters nearly without bound—most parameters are unobservable for exoplanets (Section 2).

#### 3.1.2. Plausible production rates: Source vs. sinks.

To evaluate a potential biosignature gas we must consider its source rate vs. sink rate. The dominant sink is photochemistry (Fig. 5), which is the key controlling factor for gas accumulation in an atmosphere. This is because trace gases, including biosignatures, are rapidly degraded by photochemical reactions. Gases are either directly destroyed by the host star’s incident UV radiation or readily react with other abundant photochemically produced atmospheric species, such as OH, H, and halogen radicals. A first assessment step is to calculate the gas abundance required to generate a detectable spectral feature, through forward modeling. This step naturally includes a distinguishability criterion, namely that the gas should have one or more spectral features that are distinctive compared to anticipated background gases. The second step is to evaluate the candidate biosignature gas production rates, necessary for its accumulation in an exoplanet’s atmosphere, against photochemical destruction and other sinks. A final step, if feasible, is to estimate the corresponding biomass (46). If the necessary production rates are excessively high then it is improbable for such gases to accumulate to detectable levels, or it might indicate an unrealistically large biomass. This framework intends to be pragmatic “reality check.” Admittedly, “plausible” production rate is qualitative, but we may for example, use a comparison to Earth’s production rates.

We emphasize that detectable levels that come out of computer simulations are gases with abundances of typically 1 to 100 ppm, much higher than biological or other trace gases of 1 ppt or 1 ppb typically reached in Earth’s atmosphere (e.g., ref. 5). What is important to recognize is a candidate biosignature gas must saturate any sink, not just photochemical; surface sinks as well, including water oceans. Photochemical destruction rates are driven by the UV radiation from the host star and directly set the required biological production rates (47). The need for a deep understanding of photochemistry for biosignature gas studies echoes the giant exoplanet atmosphere frontier: a merging of astronomy and a new subbranch of astrochemistry (Section 1).

### 3.2. A Growing List of Biosignature Gases.

Many gases beyond O_2_ and CH_4_ have been studied as potential biosignature gases (see ref. 48 and references therein). We present a summary in Table 1, followed by a nearly complete list of candidate biosignature gases studied to date in *SI Appendix*. A glance at Table 1 will show that there are no biosignature gases that fulfill all the criteria for robust statements about the presence of life. We omit nongas biosignatures (e.g., hazes, the red edge, algal bloom color changes, bioluminescence) as well as antibiosignatures and false negatives, since robust assessment of these is infeasible in the JWST era (Section 2).

**Table 1.** A list of potential biosignature gases with subjective evaluation

Gas	Distinctive spectral features (vs. dominant gas)	Plausible production rate	No known significant false positives (context)	Comments
O_2_	✓	✓	X	
CH_4_	✓	✓	X	
N_2_O	✓	(✓)	(✓)	
CH_3_Cl	✓	✓	(✓)	
CH_3_Br	✓	✓	(✓)	
CH_3_OH	✓	X	✓	Highly water soluble
CH_3_SH	✓	✓	X	
DMS	✓	✓	X	
PH_3_	✓	(✓)	✓	Relatively reactive
NH_3_	✓	X	✓	Highly water soluble
Isoprene	✓	X	✓	Reactive
Carbonyls	X	X	X	Highly water soluble
HCN	✓	✓	X	
NO_2_	✓	✓	X	
SF_6_	✓	X	✓	Technosignature gas
NF_3_	✓	X	✓	Technosignature gas
CFCs	✓	(✓)	✓	Technosignature gas
PFCs	✓	(✓)	✓	Technosignature gas

Reasonable production rate is subjective and may be qualified as compared to Earth values or Earth’s O_2_ production rate. See *SI Appendix* for a nearly exhaustive, referenced list of biosignature gases proposed so far.

### 3.3. JWST’s First Tentatively Claimed Biosignature Gas.

We are off to a problematic start for biosignature gases with JWST, with the potential claim of dimethyl sulfide [(CH_3_)_2_S; DMS] in the atmosphere of the sub-Neptune K2-18b (17). In this scenario, K2-18b is a presumed “Hycean World” with a H_2_-dominated atmosphere above a water ocean. The DMS detection or nondetection depends on treatment of an instrumental offset which may cause a slight discontinuity in the data. Furthermore, the tentative 2.4σ detection refers to the preference of a reference model consisting of 11 chemical species including 5 potential biosignature gases, including DMS, relative to a model with 10 chemical species of which 4 are potential biosignature gases not including DMS. That is, what is called a tentative detection hinges on a specific offset treatment and presumes that a model with 5 potential biosignatures is an appropriate reference. Regardless of offset treatment and parameter degeneracies, at face value, there is no robust statistical significance of the DMS spectral feature in the data. Instead, the detection relies on the retrieval process itself (See Section 2).

The concept of DMS in K2-18b is further explored by ref. 49 with atmosphere computer simulations for DMS and other sulfur biosignature gases across a wide range of biological fluxes and stellar UV environments. For a mixture of DMS and other sulfur gases to reach JWST detectable levels (i.e., above ~ppm levels detectable in five transits) in K2-18b, the required volatile sulfur-compound biological flux is ~20 times higher than that of DMS’ on modern Earth, as balanced against photochemical sinks.

The example of the tentative detection of DMS in K2-18b’s atmosphere is the exoplanet community’s first encounter with a biosignature gas prospect—a claim that fails all three Key Criteria above. The detection is not robust; the signal is not statistically significant (~3σ) and is sensitive to an instrumental offset. The attribution of the signal may be incorrect, as other sulfur gases share spectral features (50). Interpreting DMS as a biosignature must be confronted with potential abiotic formation scenarios (*SI Appendix*, Table S2). The DMS attribution challenges echo lessons from the initial K2-18b H_2_O detection (29, 30) later argued to be (51) and confirmed as CH_4_ (17). While higher SNR, higher spectral resolution data with broader wavelength coverage that encompass several DMS spectral features may help resolve the first two criteria, the third—interpretation—may remain unresolved due to unknown atmospheric contexts and processes.

For other suggested yet contentious biosignature gas reports, PH_3_ on Venus, CH_4_ on Mars, and HCN as a prebiotic gas on GJ 1132 b, see *SI Appendix*.

## 4. Reflecting on the Future of Biosignature Detection

We conclude with the sobering realization that with JWST we may never be able to *definitively* claim the discovery of a biosignature gas in an exoplanet atmosphere. This realization is largely motivated by the challenge of the interpretation of false positives amid the unknown planetary environments. But it is also exacerbated by the limited number of targets (Section 1), the likelihood that the spectral feature signals will be weak for small planet atmospheres (Section 1), and the limitations of the inference methods required to wrestle sparse data of a spatially and vertically unresolved planet into useful constraints on underlying planet atmosphere gas abundances (Section 2).

To confidently achieve the goal of identifying exoplanet biosignature gas candidates we should:

• Solve the effect of contamination by stellar magnetic activity;
• Discover long-period sub-Neptune targets that are colder than our current crop and use atmosphere observations to infer if any may harbor a temperate water ocean;
• Refine our atmospheric retrieval methods;
• Build our list of plausible biosignature gases readily accessible by JWST, including the gases required for context.

On the theory side, we can develop a legitimate biosignature gas assessment pathway requiring a “Deep Planet Simulator.” We may strive to develop comprehensive models for exoplanet interiors, akin to the elaborate Illustris cosmological simulation of galaxy formation (52). However, the cosmologists have a “unified theory” to test and refine, whereas the exoplanetary community is far from any unified theory of planetary habitability. By modeling from deep cores through dynamic mantles to complex atmospheres, we could seek to understand the conditions that might support identification of biosignature gases over false positives. However, unlike for galaxy formation and evolution models which were bolstered by increasing spatial resolution via new telescopes over time, there is no way to validate exoplanet interior computer models using remote sensing of spatially averaged atmospheres. As components of these models evolve and integrate experimental data, their credibility in identifying potential biosignatures will be a focal point of rigorous debate. Lab experiments need to be expanded on, as well as further synergies with the Solar System community.

Beyond JWST, we can pursue the discovery of an Earth twin around a Sun-like star using future high-contrast starlight-suppression direct imaging such as space-based mission concepts like NASA’s Habitable Worlds Observatory, Starshade (53), and the Large Interferometer for Exoplanets (LIFE) (54), or even ground-based observatories (55). A familiar scenario, with an Earth-like atmospheric mix of O_2_ and water (as opposed to a super-Earth around an M dwarf star) might yield more definitive answers, but likely will still face the wide unknowns of planetary context.

We can investigate technosignature gases—artificially produced volatiles, either intentional or accidental, by advanced civilization (56). For example, fluorine is largely avoided by life on Earth but widely used in human-made products. While technosignatures might overlap with biosignature gases if alien life uses fluorine, fluorine-based technosignatures have few, if any, false positives (*SI Appendix*, Table S2).

Ultimately, we seek fundamentally new technological approaches for exoplanet observations. Real breakthroughs will come from audacious projects like the Solar Gravitational Lens telescope, positioned 500 AU from Earth to exploit the Sun as a gravitational lens (57), or from the Starshot initiative, which envisions sending thousands of space chips with solar sails to pass by a planet and capture brief, yet potentially revealing, snapshots to send back to Earth.

The exoplanet community has come a long way in 30 y, establishing that exoplanets are common, and that rocky planets exist, including some that may have surface liquid water. We expect to find biosignature gas candidates, even if we cannot guarantee they are signs of life—an uncertainty we will have to live with for now. In the years to come, JWST will remain the flagship of this era of discovery, and will be remembered as the first telescope that set the first concrete steps toward answering the question: Are we alone?

## Supplementary Material

Appendix 01 (PDF)

## Data Availability

All study data are included in the article and/or *SI Appendix*.

## Footnotes

^*^The habitable zone is the region around a star in which a planet may maintain liquid water on its surface, based on its surface temperature for a planet atmosphere heated by the radiation from the host star.
