# Supplementary material for: Prospects for detecting signs of life on exoplanets in the JWST era
Source: Proc Natl Acad Sci U S A. 2025 Sep 22;122(39):e2416188122. doi: 10.1073/pnas.2416188122 (PMC12501172; doi:10.1073/pnas.2416188122)
Supplement: Supplementary file 1 — Appendix 01 (PDF) [file pnas.2416188122.sapp.pdf]

PNAS

Supplementary Information for

Prospects for Detecting Signs of Life on Exoplanets in the JWST Era.

Sara Seager[1,2,3,*], Luis Welbanks[4], Lucas Ellerbroek[5], William Bains[6], Janusz J. Petkowski[7,8]

[1] Department of Earth, Atmospheric and Planetary Sciences, Massachusetts Institute of Technology, Cambridge, MA, USA
[2] Department of Physics, Massachusetts Institute of Technology, Cambridge, MA, USA
[3] Department of Aeronautical and Astronautical Engineering, Massachusetts Institute of Technology, Cambridge, MA, USA
[4] School of Earth and Space Exploration, Arizona State University, Tempe, AZ, USA
[5] Department of Astrophysics / IMAPP, Radboud University, PO Box 9010, 6500 GL, Nijmegen, The Netherlands
[6] School of Physics and Astronomy, Cardiff University, 4 The Parade, Cardiff CF24 3AA, UK
[7] JJ Scientific, Warsaw, Mazowieckie, Poland
[8] Faculty of Environmental Engineering, Wroclaw University of Science and Technology, 50-370 Wroclaw, Poland

*Correspondence: Sara Seager
Email: seager@mit.edu

This PDF file includes:

Supplementary text
Tables S1 to S2
SI References

Supplementary Information Text

1. Biosignature Gas Candidate Detections Preceding the JWST Era.

There are few reports of biosignature gases detections that precede the coming era of the JWST exoplanet atmosphere studies – the reports of phosphine (PH_3) on Venus (1), methane (CH_4) on Mars (2) and hydrogen cyanide (HCN) and CH_4, as prebiotic molecules, on GJ 1132b (3). All three cases exemplify the difficulty of such observations, and the inherent uncertainty of data interpretation that will challenge the JWST search for genuine signs of life. We summarize these examples briefly below.

The detection of an atmosphere on a rocky Earth-size exoplanet, GJ 1132b has been reported in 2021. The Hubble WFC3 infrared atmospheric transmission spectrum has been claimed to show spectral signatures of aerosol scattering, HCN, and CH_4 gas in a low mean molecular weight atmosphere (3). Other groups that reanalyzed the same Hubble data find a featureless spectrum, with no evidence of an atmosphere (4, 5). The initial observations of GJ 1132b have also recently been followed up with JWST. The thermal emission measurements of GJ 1132b with the Mid-Infrared Instrument Low-Resolution Spectrometer (MIRI), in 5–12 μm, support the conclusion that GJ 1132b likely does not have a significant atmosphere (6).

The search for biosignature gases in our on solar system is also controversial. The detection of CH_4 on Mars was first reported in 2004 from both a ground-based telescope (2) and by the Mars Express Orbiter (7). Both found a weak signal with small values, 10 ± 3 ppb and 10 ± 5 ppb respectively. The initial observations were followed by in situ detection by the TLS component of the SAM instrument on the Mars Curiosity Rover (e.g. (8, 9)).

There is a continuing discussion on the existence of CH_4 on Mars. The Trace Gas Orbiter (TGO) does not observe methane and set a detection limit of 0.05 ppb despite hundreds of observations (10). Criticism that the ground-based detections by (11) were simply a detection of the $^{13}CH_4$ isotope in Earth's atmosphere (12) were later refuted (13). The in situ TLS/SAM findings of seasonal methane (9) come from an instrument designed to avoid contamination (14), but have been argued to be contamination (15) or noise (16).

Methane is not expected to be present on Mars because it is a reduced gas and the Martian atmosphere and surface are oxidizing environments. The presence of CH_4 would indicate an unusual geochemistry or leave the possibility for the presence of life. The relatively short ~300 year photochemical lifetime of CH_4 means that it must have a current-day (or very recent) source. This source, if present, is unknown. For a summary of the Martian CH_4 debate see (14, 17).

Phosphine (PH_3) gas in the Venus atmosphere was reported at a few ppb levels in 2020 (1) and followed up by evidence of PH_3 from a data reanalysis of NASA's 1978 Pioneer Venus Probe's mass spectrometer (18). While additional astronomical observations have supported the presence of PH_3 (19), the majority do not detect the signal at all (20–22), instead ascribing the result to statistical or processing errors (23–25). Some others do recover the signal and propose gas attribution to SO_2 rather than PH_3 (26, 27).

On Venus and Earth PH_3 should not be present due to the oxidizing environment and short PH_3 photochemical lifetime. On Earth, PH_3 only has biological or industrial sources (e.g. (28)). Work has largely demonstrated that known abiotic chemistry—lightning, volcanoes, meteoritic delivery—in the Venus environment does not produce the required amounts of PH_3 to explain claimed levels (e.g. (29, 30)), though future research may find viable pathways due to unknown PH_3 chemistry (Table S2). For a recent review of observational and interpretation claims and refutations, see (31).

That the detection of methane on Mars is still not accepted 20 years after its first report, despite detections from Earth, Mars orbit, and Mars highlights the challenges in addressing trace gases for questions [1] and [2], let alone the more difficult question [3]. Here questions are defined in the main manuscript Section 4. Similarly, the ongoing debate on the presence of PH_3 on Venus shows that clear

answers to questions [1] and [2] likely will not be resolved without a dedicated space mission with an in-situ measurement capability. The answer to question [3] also remains inconclusive (Table S2).

Although both Mars and Venus are challenging environments for which to imagine life's existence—Mars life needing to be confined subsurface and Venus' to temperate cloud layers composed of sulfuric acid—both are neighboring planets which we know far more about than we will know about any exoplanet. One may have thought if there ever was a place where it would be possible to confirm a biosignature, it would be a planet next door, especially as we can and have sent in situ probes to physically sample the environment. Sampling an exoplanet remains in the realm of science fiction.

Table S1. Selected biosignature gases and their JWST observational prospects. The table shows selected JWST observational prospects focusing on the diverse stellar and planetary scenarios. We only show published computer-simulated predictions, not the proposed, or approved, JWST observational campaigns.

**Biosignature Gas**	**Stellar Type**	**Planet Type**	**Atm. Type**	**JWST Detectable Flux or Abundance / at λ (μm)**	**JWST Observation Time**
O_2/O_3	Trappist-1 (M8V)	Rocky Earth size (Trappist-1e)	Archean Earth	Not detectable (32–37)	N/A
CH_4/CO_2 pair	Trappist-1 (M8V)	Rocky Earth size (Trappist-1e)	Archean Earth	Archean-Earth-like CH_4 levels	10 transits (35, 38, 39)
CH_4/CO_2 pair	LHS 1140 (M4.5)	Sub Neptune (LHS 1140 b)	H_2	? / NIRSpec [1-5 μm]	1 transit (40)
N_2O	Trappist-1 (M8V)	Rocky Earth size (Trappist-1e)	N_2-O_2	10–100 Tmol yr^{-1} / 2.9 μm	detectable "within [JWST] mission lifetime" (41)
N_2O	LHS 1140 (M4.5)	Sub Neptune (LHS 1140 b)	H_2	? / NIRSpec [1-5 μm]	10–50 transits (40–200 hours) (40)
CH_3Cl/CH_3Br pair	Trappist-1 (M8V)	Rocky Earth-size (Trappist-1e)	N_2-O_2	10^{12} molecules cm^{-2} s^{-1} / MIRI-LRS [5-14 μm]	10 transits (42)
CH_3Cl	LHS 1140 (M4.5)	Sub Neptune (LHS 1140 b)	H_2	? / NIRSpec [1-5 μm]	10–50 transits (40–200 hours) (40)
CH_3OH	M5V (based on GJ 876)	Rocky super Earth (1.5 R_{Earth}, 5 M_{Earth})	H_2	10 ppm / G395M [2.87-5.14 μm] and MIRI-LRS [5-14 μm]	20 transits (43)
CH_3SH	Trappist-1 (M8V)	Rocky Earth size (Trappist-1e)	Archean Earth	30 x Earth flux rate / NIRSpec [1-5 μm]	50 transits (35)
DMS	K2-18 (M2.8)	Sub Neptune (K2-18b)	H_2	20 x Earth flux rate / MIRI-LRS [5-14 μm]	5 transits (44)
DMS	Trappist-1 (M8V)	Rocky Earth size (Trappist-1e)	Archean Earth	30 x Earth flux rate / NIRSpec [1-5 μm]	94 transits (35)
PH_3	Active M-dwarf	Rocky super Earth (1.75 R_{Earth}, 10 M_{Earth})	H_2	4 ppm / 7.8-11.5 μm in emission	52 hours (28)
PH_3	Active M-dwarf	Rocky super Earth (1.75 R_{Earth}, 10 M_{Earth})	CO_2	310 ppm / 7.8-11.5 μm in emission	48 hours (28)
PH_3	LHS 1140 (M4.5)	Sub Neptune (LHS 1140 b)	H_2	10^{10} molecules cm^{-2} s^{-1} / NIRSpec [1-5 μm]	10–50 transits (40–200 hours) (40)
NH_3	M5V	Rocky super Earth (1.75 R_{Earth}, 10 M_{Earth})	H_2	5 ppm NIRSpec [1-5 μm] and MIRI-LRS [5-14 μm]	80 transits (45)

NH_3	M4V (based on GJ 1132)	Rocky super Earth (1.75 R_{Earth}, 10 M_{Earth})	H_2	14 ppm / MIRI-LRS [5-14 µm]	3 transits (46)
NH_3	LHS 1140 (M4.5)	Sub Neptune (LHS 1140 b)	H_2	8.4×10^{10} molecules cm^{-2} s^{-1} / NIRSpec [1-5 µm]	10–50 transits (40–200 hours) (40)
Isoprene	M5V	Rocky super Earth (1.75 R_{Earth}, 10 M_{Earth})	H_2	3×10^{13} molecules cm^{-2} s^{-1} / NIRSpec [1-5 µm] and MIRI	20 transits (47)
Formaldehyde (CH_2O) and other carbonyls	M4V (based on GJ 876)	Rocky super Earth (1.75 R_{Earth}, 10 M_{Earth})	H_2	Not detectable (48)	N/A
Formaldehyde (CH_2O) and other carbonyls	M4V (based on GJ 1132)	Rocky super Earth (1.75 R_{Earth}, 10 M_{Earth})	H_2	3.1 ppm / MIRI-LRS [5-14 µm]	3 transits (46)
HCN	M4V (based on GJ 1132)	Rocky super Earth (1.75 R_{Earth}, 10 M_{Earth})	H_2	1.7 ppm / G395M [2.87-5.14 µm]	3 transits (46)
NO_2	M dwarf	Rocky Earth size	N_2	20 x present Earth's atm. abundance	500 hours (49)
SF_6	M5V (based on GJ 876)	Rocky super Earth (1.5 R_{Earth}, 5 M_{Earth})	H_2	1 ppm / 9-12 µm	Tens of transits (50)
SF_6	Trappist-1 (M8V)	Rocky Earth size (Trappist-1f)	N_2-O_2	100 ppm / MIRI-LRS [5-14 µm]	19 transits (51)
NF_3	M5V (based on GJ 876)	Rocky super Earth (1.5 R_{Earth}, 5 M_{Earth})	H_2	1 ppm / 9-12 µm	Tens of transits (50)
NF_3	Trappist-1 (M8V)	Rocky Earth size (Trappist-1f)	N_2-O_2	100 ppm / MIRI-LRS [5-14 µm]	16 transits (51)
CFCs	White dwarf	Rocky Earth size	N_2	10 x present Earth's atm. abundance	30-40 hours (52)
CFCs	M dwarf	Rocky Earth size	N_2	>1 ppb	100-300 hours (53)
PFCs	Trappist-1 (M8V)	Rocky Earth size (Trappist-1f)	N_2-O_2	100 ppm / MIRI-LRS [5-14 µm]	5 transits (51)

Table S2. False positive assessment of biosignature gases.

Biosig. Gas	Proposed Abiotic Source
O_2/O_3	Massive O_2 atmospheres (with partial pressures $pO_2 \geq 10$ bar) are postulated to exist via abiotic accumulation on M-dwarf-hosted exoplanets. The main proposed mechanism is XUV-driven photodissociation and escape during the extended pre-main sequence runaway greenhouse phase (54). Terrestrial planets orbiting any star type may develop O_2-dominated atmospheres as a result of H_2O photolysis due to inefficient cold trap (55). Massive O_2 atmospheres are thought to only form abiotically, and can be identified by the presence of O_2-O_2 dimers (56, 57). Abiotic O_2 and O_3 can also form through planetary photochemistry of CO_2 (58, 59). Conditions for O_2 false positives on habitable zone planets around Sun-like stars have also been explored (60).
CH_4	Methane can be produced geologically, e.g., in the serpentinization reaction but large amounts of CH_4 are unlikely to have volcanic origin (61). CH_4 can also be produced by impacts (62, 63). Its potential as a biosignature highly depends on the stellar and planetary context (64).
N_2O	Significant abiotic sources of N_2O are limited. Potential abiotic sources like reduction of nitric oxide (NO) by ferrous iron (chemidenitrification), lightning, volcanic activity, or photochemical reduction of NO can be ruled out or identified by relevant stellar and planetary context (41).
CH_3Cl	Meteoritic infall and volcanism could be the source of small amounts of abiotic CH_3Cl (42).
CH_3Br	Meteoritic infall and volcanism could be the source of small amounts of abiotic CH_3Br (42).
CH_3OH	There are no known significant abiotic CH_3OH sources on terrestrial planets (43).
CH_3SH	Geological and hydrothermal sources are unlikely to generate CH_3SH in significant amounts (65). Laboratory photochemical experiments demonstrate the abiotic production of organosulfur gases (66).
DMS	Geological and hydrothermal sources are unlikely to generate DMS in significant amounts (65). Laboratory photochemical experiments demonstrate the abiotic production of organosulfur gases (66). DMS may also be produced abiotically in cometary matter (67) and the interstellar medium (68).
PH_3	No known significant abiotic source on rocky exoplanets (28). In the context of Venus: no significant conventional abiotic sources known (29, 31). Volcanoes are an inefficient source of PH_3 (30). Other potential abiotic sources like reduction with Fe-rich minerals (69) or synthesis over acidic dust in Venus' atmosphere await confirmation (70).
NH_3	No known significant abiotic source on rocky exoplanets (45). Volcanic, hydrothermal, and other processes (e.g. nitrogen photoreduction on TiO_2 containing sands) are too inefficient to result in a detectable NH_3 (45).
Isoprene	Isoprene (C_5H_8) has no known false positive sources (47).
Carbonyls	Formation of formaldehyde (CH_2O) via photochemical processes is potentially possible (46, 71) although its accumulation in the atmosphere to significant amounts is unlikely (48).
HCN	Hydrogen cyanide (HCN) can be produced via UV photochemistry (46, 72–75), surface hydrothermal systems (76), impacts (77, 78), and lightning (79).
NO_2	Produced via lightning from atmospheric N_2 and CO_2 (65).
SF_6	Sulfur hexafluoride (SF_6) has very few false positives. Trace amounts of SF_6 are associated with volcanic activity (80–82). SF_6 can be released from fluorite minerals (83). H-depleted planets could potentially produce abiotic SF_6 in greater amounts than on Earth (50).
NF_3	Nitrogen trifluoride (NF_3) has no known significant false positive sources. NF_3 is not known to be released from any fluorine-containing minerals apart from a single exception of purple fluorite (WF) (83).
CFCs	Various CFCs can be produced in trace amounts by volcanoes (e.g. (84, 85)) or can be detected in trace amounts in fluorite minerals (83).
PFCs	Abiotic CF_4 is produced geologically in trace amounts (e.g. (81–83)).

Supplementary References

1. J. S. Greaves, *et al.*, Phosphine gas in the cloud decks of Venus. *Nat. Astron.* **5**, 655–664 (2021).
2. V. A. Krasnopolsky, J. P. Maillard, T. C. Owen, Detection of methane in the martian atmosphere: Evidence for life? *Icarus* **172**, 537–547 (2004).
3. M. R. Swain, *et al.*, Detection of an atmosphere on a rocky exoplanet. *Astron. J.* **161**, 213 (2021).
4. L. V Mugnai, *et al.*, ARES.* V. No evidence for molecular absorption in the HST WFC3 spectrum of GJ 1132 b. *Astron. J.* **161**, 284 (2021).
5. J. E. Libby-Roberts, *et al.*, The featureless HST/WFC3 transmission spectrum of the rocky exoplanet GJ 1132b: no evidence for a cloud-free primordial atmosphere and constraints on starspot contamination. *Astron. J.* **164**, 59 (2022).
6. Q. Xue, *et al.*, JWST Thermal Emission of the Terrestrial Exoplanet GJ 1132b. *Astrophys. J. Lett.* **973**, L8 (2024).
7. V. Formisano, S. Atreya, T. Encrenaz, N. Ignatiev, M. Giuranna, Detection of methane in the atmosphere of Mars. *Science (80-.).* **306**, 1758–1761 (2004).
8. C. R. Webster, *et al.*, Mars methane detection and variability at Gale crater. *Science (80-.).* **347**, 415–417 (2015).
9. C. R. Webster, *et al.*, Background levels of methane in Mars' atmosphere show strong seasonal variations. *Science (80-.).* **360**, 1093–1096 (2018).
10. O. Korablev, *et al.*, No detection of methane on Mars from early ExoMars Trace Gas Orbiter observations. *Nature* **568**, 517–520 (2019).
11. M. J. Mumma, *et al.*, Strong Release of Methane. *Science (80-.).* **323**, 1041–1045 (2009).
12. K. Zahnle, R. S. Freedman, D. C. Catling, Is there methane on Mars? *Icarus* **212**, 493–503 (2011).
13. G. L. Villanueva, *et al.*, A sensitive search for organics (CH4, CH3OH, H2CO, C2H6, C2H2, C2H4), hydroperoxyl (HO2), nitrogen compounds (N2O, NH3, HCN) and chlorine species (HCl, CH3Cl) on Mars using ground-based high-resolution infrared spectroscopy. *Icarus* **223**, 11–27 (2013).
14. C. R. Webster, *et al.*, Day-night differences in Mars methane suggest nighttime containment at Gale crater. *Astron. Astrophys.* **650**, A166 (2021).
15. B. K. Zahnle, Play it again, SAM. *Science (80-.).* **347**, 370–371 (2015).
16. E. Gillen, P. B. Rimmer, D. C. Catling, Statistical analysis of Curiosity data shows no evidence for a strong seasonal cycle of Martian methane. *Icarus* **336**, 113407 (2020).
17. J. L. Grenfell, *et al.*, Atmospheric processes affecting methane on Mars. *Icarus* **382**, 114940 (2022).
18. R. Mogul, S. S. Limaye, M. J. Way, J. A. Cordova, Venus' Mass Spectra Show Signs of Disequilibria in the Middle Clouds. *Geophys. Res. Lett.*, e2020GL091327 (2021).
19. D. L. Clements, Venus Phosphine: Updates and lessons learned. *arXiv Prepr.*

arXiv2409.13438 (2024).

20. L. Trompet, *et al.*, Phosphine in Venus' atmosphere: Detection attempts and upper limits above the cloud top assessed from the SOIR/VEx spectra. *Astron. Astrophys.* **645**, L4 (2020).

21. T. Encrenaz, *et al.*, A stringent upper limit of the PH3 abundance at the cloud top of Venus. *Astron. Astrophys.* **643**, L5 (2020).

22. M. A. Cordiner, *et al.*, Phosphine in the Venusian Atmosphere: A Strict Upper Limit from SOFIA GREAT Observations. *Geophys. Res. Lett.*, e2022GL101055 (2022).

23. A. B. Akins, A. P. Lincowski, V. S. Meadows, P. G. Steffes, Complications in the ALMA Detection of Phosphine at Venus. *Astrophys. J. Lett.* **907**, L27 (2021).

24. M. A. Thompson, The statistical reliability of 267-GHz JCMT observations of Venus: no significant evidence for phosphine absorption. *Mon. Not. R. Astron. Soc. Lett.* **501**, L18–L22 (2021).

25. I. A. G. Snellen, L. Guzman-Ramirez, M. R. Hogerheijde, A. P. S. Hygate, F. F. S. van der Tak, Re-analysis of the 267 GHz ALMA observations of Venus-No statistically significant detection of phosphine. *Astron. Astrophys.* **644**, L2 (2020).

26. G. L. Villanueva, *et al.*, No evidence of phosphine in the atmosphere of Venus from independent analyses. *Nat. Astron.* **5**, 631–635 (2021).

27. A. P. Lincowski, *et al.*, Claimed detection of PH3 in the clouds of Venus is consistent with mesospheric SO2. *Astrophys. J. Lett.* **908**, L44-52 (2021).

28. C. Sousa-Silva, *et al.*, Phosphine as a biosignature gas in exoplanet atmospheres. *Astrobiology* **20**, 235–268 (2020).

29. W. Bains, *et al.*, Phosphine on Venus Cannot be Explained by Conventional Processes. *Astrobiology* **21**, 1277–1304 (2021).

30. W. Bains, *et al.*, Constraints on the production of phosphine by Venusian volcanoes. *Universe* **8**, 54 (2022).

31. W. Bains, *et al.*, Source of phosphine on Venus—An unsolved problem. *Front. Astron. Sp. Sci.* **11**, 1372057 (2024).

32. F. Wunderlich, *et al.*, Detectability of atmospheric features of Earth-like planets in the habitable zone around M dwarfs. *Astron. Astrophys.* **624**, A49 (2019).

33. M. T. Gialluca, T. D. Robinson, S. Rugheimer, F. Wunderlich, Characterizing atmospheres of transiting Earth-like exoplanets orbiting M dwarfs with James Webb Space Telescope. *Publ. Astron. Soc. Pacific* **133**, 54401 (2021).

34. F. Wunderlich, *et al.*, Distinguishing between Wet and Dry Atmospheres of TRAPPIST-1 e and f. *Astrophys. J.* **901**, 126 (2020).

35. V. S. Meadows, A. P. Lincowski, J. Lustig-Yaeger, The Feasibility of Detecting Biosignatures in the TRAPPIST-1 Planetary System with JWST. *Planet. Sci. J.* **4**, 192 (2023).

36. J. Lustig-Yaeger, V. S. Meadows, A. P. Lincowski, The detectability and characterization of the TRAPPIST-1 exoplanet atmospheres with JWST. *Astron. J.* **158**, 27 (2019).

37. D. Pidhorodetska, T. J. Fauchez, G. L. Villanueva, S. D. Domagal-Goldman, R. K. Kopparapu, Detectability of molecular signatures on TRAPPIST-1e through transmission spectroscopy simulated for future space-based observatories. *Astrophys. J. Lett.* **898**, L33 (2020).

38. J. Krissansen-Totton, R. Garland, P. Irwin, D. C. Catling, Detectability of biosignatures in anoxic atmospheres with the James Webb Space Telescope: a TRAPPIST-1e case study. *Astron. J.* **156**, 114 (2018).

39. T. Mikal-Evans, Detecting the proposed CH4–CO2 biosignature pair with the James Webb Space Telescope: TRAPPIST-1e and the effect of cloud/haze. *Mon. Not. R. Astron. Soc.* **510**, 980–991 (2022).

40. F. Wunderlich, *et al.*, Detectability of biosignatures on LHS 1140 b. *Astron. Astrophys.* **647**, A48 (2021).

41. E. W. Schwieterman, *et al.*, Evaluating the plausible range of N2O biosignatures on exo-earths: An integrated biogeochemical, photochemical, and spectral modeling approach. *Astrophys. J.* **937**, 109 (2022).

42. M. Leung, E. W. Schwieterman, M. N. Parenteau, T. J. Fauchez, Alternative methylated biosignatures. I. Methyl bromide, a capstone biosignature. *Astrophys. J.* **938**, 6 (2022).

43. J. Huang, S. Seager, J. J. Petkowski, Z. Zhan, S. Ranjan, Methanol—A Poor Biosignature Gas in Exoplanet Atmospheres. *Astrophys. J.* **933**, 6 (2022).

44. S.-M. Tsai, H. Innes, N. F. Wogan, E. W. Schwieterman, Biogenic sulfur gases as biosignatures on temperate sub-Neptune waterworlds. *Astrophys. J. Lett.* **966**, L24 (2024).

45. J. Huang, S. Seager, J. J. Petkowski, S. Ranjan, Z. Zhan, Assessment of ammonia as a biosignature gas in exoplanet atmospheres. *Astrobiology* **22**, 171–191 (2022).

46. A. B. Claringbold, P. B. Rimmer, S. Rugheimer, O. Shorttle, Prebiosignature molecules can be detected in temperate exoplanet atmospheres with JWST. *Astron. J.* **166**, 39 (2023).

47. Z. Zhan, *et al.*, Assessment of Isoprene as a Possible Biosignature Gas in Exoplanets with Anoxic Atmospheres. *Astrobiology* **21**, 765–792 (2021).

48. Z. Zhan, J. Huang, S. Seager, J. J. Petkowski, S. Ranjan, Organic Carbonyls Are Poor Biosignature Gases in Exoplanet Atmospheres but May Generate Significant CO. *Astrophys. J.* **930**, 133 (2022).

49. R. Kopparapu, G. Arney, J. Haqq-Misra, J. Lustig-Yaeger, G. Villanueva, Nitrogen dioxide pollution as a signature of extraterrestrial technology. *Astrophys. J.* **908**, 164 (2021).

50. S. Seager, *et al.*, Fully fluorinated non-carbon compounds NF3 and SF6 as ideal technosignature gases. *Sci. Rep.* **13**, 13576 (2023).

51. E. W. Schwieterman, *et al.*, Artificial Greenhouse Gases as Exoplanet Technosignatures. *Astrophys. J.* **969**, 20 (2024).

52. H. W. Lin, G. G. Abad, A. Loeb, Detecting industrial pollution in the atmospheres of earth-like exoplanets. *Astrophys. J. Lett.* **792**, L7 (2014).

53. J. Haqq-Misra, *et al.*, Detectability of Chlorofluorocarbons in the Atmospheres of

Habitable M-dwarf Planets. *Planet. Sci. J.* **3**, 60 (2022).

54. R. Luger, R. Barnes, Extreme water loss and abiotic O2 buildup on planets throughout the habitable zones of M dwarfs. *Astrobiology* **15**, 119–143 (2015).

55. R. Wordsworth, R. Pierrehumbert, Abiotic Oxygen-dominated Atmospheres on Terrestrial Habitable Zone Planets. *Astrophys. J.* **785**, L20 (2014).

56. A. Misra, V. Meadows, M. Claire, D. Crisp, Using dimers to measure biosignatures and atmospheric pressure for terrestrial exoplanets. *Astrobiology* **14**, 67–86 (2014).

57. E. W. Schwieterman, *et al.*, Identifying Planetary Biosignature Impostors: Spectral Features of Co and O(4) Resulting From Abiotic O(2)/O(3) Production. *Astrophys. journal. Lett.* **819**, L34 (2016).

58. V. S. Meadows, *et al.*, Exoplanet Biosignatures: Understanding Oxygen as a Biosignature in the Context of Its Environment. *Astrobiology* **18**, 630–662 (2018).

59. V. S. Meadows, Reflections on O2 as a biosignature in exoplanetary atmospheres. *Astrobiology* **17**, 1022–1052 (2017).

60. J. Krissansen-Totton, J. J. Fortney, F. Nimmo, N. Wogan, Oxygen False Positives on Habitable Zone Planets Around Sun-Like Stars. *AGU Adv.* **2**, e2020AV000294 (2021).

61. N. Wogan, J. Krissansen-Totton, D. C. Catling, Abundant atmospheric methane from volcanism on terrestrial planets is unlikely and strengthens the case for methane as a biosignature. *Planet. Sci. J.* **1**, 58 (2020).

62. M. E. Kress, C. P. McKay, Formation of methane in comet impacts: implications for Earth, Mars, and Titan. *Icarus* **168**, 475–483 (2004).

63. Y. Sekine, S. Sugita, T. Kadono, T. Matsui, Methane production by large iron meteorite impacts on early Earth. *J. Geophys. Res. Planets* **108** (2003).

64. M. A. Thompson, J. Krissansen-Totton, N. Wogan, M. Telus, J. J. Fortney, The case and context for atmospheric methane as an exoplanet biosignature. *Proc. Natl. Acad. Sci.* **119**, e2117933119 (2022).

65. S. Seager, M. Schrenk, W. Bains, An astrophysical view of Earth-based metabolic biosignature gases. *Astrobiology* **12**, 61–82 (2012).

66. N. W. Reed, *et al.*, Abiotic Production of Dimethyl Sulfide, Carbonyl Sulfide, and Other Organosulfur Gases via Photochemistry: Implications for Biosignatures and Metabolic Potential. *Astrophys. J. Lett.* **973**, L38 (2024).

67. N. Hänni, *et al.*, Evidence for Abiotic Dimethyl Sulfide in Cometary Matter. *Astrophys. J.* **976**, 74 (2024).

68. M. Sanz-Novo, *et al.*, On the abiotic origin of dimethyl sulfide: discovery of DMS in the Interstellar Medium. *arXiv Prepr. arXiv2501.08892* (2025).

69. T. Feng, M. A. Pasek, Phosphorus acid volatility in Venus's atmosphere and its reduction with iron-rich minerals in *2024 Astrobiology Science Conference*, (AGU, 2024).

70. K. Mráziková, *et al.*, A Novel Abiotic Pathway for Phosphine Synthesis over Acidic Dust in Venus' Atmosphere. *Astrobiology* **24**, 407–422 (2024).

71. J. P. Pinto, G. R. Gladstone, Y. L. Yung, Photochemical production of formaldehyde in Earth's primitive atmosphere. *Science (80-.).* **210**, 183–185 (1980).

72. P. B. Rimmer, S. Rugheimer, Hydrogen cyanide in nitrogen-rich atmospheres of rocky exoplanets. *Icarus* **329**, 124–131 (2019).

73. B. K. D. Pearce, P. W. Ayers, R. E. Pudritz, A consistent reduced network for HCN chemistry in early earth and Titan atmospheres: Quantum calculations of reaction rate coefficients. *J. Phys. Chem. A* **123**, 1861–1873 (2019).

74. F. Tian, J. F. Kasting, K. Zahnle, Revisiting HCN formation in Earth's early atmosphere. *Earth Planet. Sci. Lett.* **308**, 417–423 (2011).

75. K. J. Zahnle, Photochemistry of methane and the formation of hydrocyanic acid (HCN) in the Earth's early atmosphere. *J. Geophys. Res. Atmos.* **91**, 2819–2834 (1986).

76. P. B. Rimmer, O. Shorttle, A Surface Hydrothermal Source of Nitriles and Isonitriles. *Life* **14**, 498 (2024).

77. M. Ferus, *et al.*, High energy radical chemistry formation of HCN-rich atmospheres on early Earth. *Sci. Rep.* **7**, 6275 (2017).

78. N. F. Wogan, D. C. Catling, K. J. Zahnle, R. Lupu, Origin-of-life Molecules in the Atmosphere after Big Impacts on the Early Earth. *Planet. Sci. J.* **4**, 169 (2023).

79. A. Ardaseva, *et al.*, Lightning Chemistry on Earth-like Exoplanets. *arXiv* **470**, 187–196 (2017).

80. E. Busenberg, L. N. Plummer, Dating young groundwater with sulfur hexafluoride: Natural and anthropogenic sources of sulfur hexafluoride. *Water Resour. Res.* **36**, 3011–3030 (2000).

81. J. Harnisch, A. Eisenhauer, Natural CF4 and SF6 on Earth. *Geophys. Res. Lett.* **25**, 2401–2404 (1998).

82. D. A. Deeds, *et al.*, Evidence for crustal degassing of CF4 and SF6 in Mojave Desert groundwaters. *Geochim. Cosmochim. Acta* **72**, 999–1013 (2008).

83. J. Harnisch, M. Frische, R. Borchers, A. Eisenhauer, A. Jordan, Natural fluorinated organics in fluorite and rocks. *Geophys. Res. Lett.* **27**, 1883–1886 (2000).

84. A. Jordan, J. Harnisch, R. Borchers, F. Le Guern, H. Shinohara, Volcanogenic halocarbons. *Environ. Sci. Technol.* **34**, 1122–1124 (2000).

85. V. A. Isidorov, I. G. Zenkevich, B. V Ioffe, Volatile organic compounds in solfataric gases. *J. Atmos. Chem.* **10**, 329–340 (1990).
